# Neuroimaging Features and Associated Factors in Multiple Sclerosis Patients: A Perspective from a Private Care Center in Addis Ababa, Ethiopia

**DOI:** 10.4314/ejhs.v31i5.17

**Published:** 2021-09

**Authors:** Biniyam A Ayele, Mehila Z Wuhib, Betesaida G Zenebe, Yared Z Zewde, Yonas T Wolde, Guta Z Metaferia

**Affiliations:** 1 Assistant Professor of Neurology, Department of Neurology College of Health Sciences, Addis Ababa University, Addis Ababa, Ethiopia; 2 Yehuleshet Specialty Clinic, Honorary Assistant Professor of Neurology; 3 Yehuleshet Specialty Clinic, Addis Ababa, Ethiopia; 4 Assistant Professor of Radiology, Neuroradiologist Department of Radiology, St.Paul Millennium Medical College, Addis Ababa, Ethiopia

**Keywords:** Multiple sclerosis, MS-related lesions, magnetic resonance imaging, Dawson's finger projections, Ethiopia

## Abstract

**Background:**

Brain and spine magnetic resonance image (MRI) have an invaluable importance in diagnosing multiple sclerosis (MS) in low prevalence countries such as Ethiopia. The objective of our study was to characterize the neuroimaging features and associated factors in Multiple sclerosis patients in Addis Ababa, Ethiopia.

**Method:**

A cross-sectional observational study was conducted in 30 multiple sclerosis patients at Yehuleshet Specialty Clinic, Addis Ababa, Ethiopia. Both descriptive and analytical statistics were used to analyze the data.

**Results:**

We have enrolled 30 patients with confirmed multiple sclerosis and clinically isolate syndrome. The mean age was 34.7 years (1SD=8.9). Female accounted 86.7%. The mean duration of illness was 3.4 years (1SD=3.1) (range: 1 – 11 years). Relapsing and remitting variant was the commonest sub type (66.7%). Alcohol use and head injury were the commonest identified risk factors reported by the patients. Classical radiological features of MS such as white matter lesions involving juxtacortical, U-fiber, corpus callosum (CC), and Dawson's finger projections pattern were observed in 46.7%, 23.3%, 70%, and 40% respectively. Cervical and thoracic cords were affected in 40% and 6.7% respectively. Global cortical and CC atrophy was observed in 16.7% and 6.7% respectively. Advanced age was associated with lesions of corpus callosum when adjusted for duration of illness and history of head injury (AOR 1.13, 95% CI 1.01–1.28, p=0.04).

**Conclusion:**

Typical neuroimaging features of MS were prevalent among Ethiopian MS patients. Age was an independent predictor of lesions involving corpus callosum. Global cortical atrophy was common among Ethiopian MS patients.

## Introduction

Diagnosis of multiple sclerosis is primarily clinical and augmented by neuroradiological findings. Revised 2017 McDonald criteria is the most common diagnostic tool utilized for clinical and research purposes (1). Multiple sclerosis is considered to be a rare neurological disorder for those living around tropical zone, especially sub Saharan Africa (SSA), because of latitude effect (2–4). Therefore, ancillary augmenting clinical diagnosis of MS with imaging modalities such as brain and spinal cord MRI would have a vital importance for clinicians suspecting MS in less prevalent region such as Ethiopia. (5–8). As one of SSA country and its location on the tropics, MS is rarely considered by most clinicians in Ethiopia (9). This is evidenced by the significant paucity of MS-related studies in the country. Thus far, there are no published studies related to MS except, a single case of MS reported by Redda TH 1985 (10). However, in the past two decades, relative improvement in the availability of advanced imaging modalities such as magnetic resonance imaging and evoked potentials resulted in significant improvement in the diagnosis of MS and other related demyelinating central nervous system (CNS) disorders. Therefore, it's imperative to characterize the MRI features of Ethiopian multiple sclerosis patients. Furthermore, it's important to compare our findings with similar studies in the western countries in order to understand tropical MS better.

The radiological hallmark of MS is focal area demyelination within the CNS, sparing the peripheral nervous system (PNS). This demyelination is due to local inflammation in response to destruction of oligodendrocyte (6). The demyelination tends to occur in a perivenous location, and is typically distributed around the ventricles (periventricular), adjacent to the cortex (juxtacortical) infratentorial (brainstem and cerebellum), within the spinal cord, and involving the subcortical U-fibers and corpus callosum (1,5,11–14). Furthermore, understanding the pattern of imaging hallmarks of MS such as Dawson's finger projection (DFP) in Ethiopian MS patients would have a great additive effect in improving diagnostic accuracy for the practicing clinicians in the country. Dawson's finger projection is one of the many ventricle-based MS lesions, in which a wave of demyelinating finger-like projections from the ventricular lining funneling towards corpus callosum (11,15,16). The objective of our study was to characterize the neuroimaging features and associated factors in multiple sclerosis patients in Addis Ababa, Ethiopia.

## Materials and Methods

**Study objective and study setting**: The aim of this study was to characterize the brain and spinal cord MRI features of thirty MS patients. The study was conducted at the outpatient neurology clinics of Yehuleshet Specialty Clinic (YSC) in Addis Ababa, Ethiopia. Yehuleshet Specialty Clinic is a specialty clinic located at the heart of Addis Ababa with high neurology case load and well equipped with necessary investigations such as: 0.35 Tesla Magnetic resonance image (MRI), Electroencephalogram (EEG), Electromyography (EMG), Nerve conduction study (NCS), and visual evoked potential (VEP). On average day the clinic is visited by 115 – 120 new and follows up adult with neurological disorders. Similarly, on average daily 30 – 40 new and follow up pediatrics patients with neurological disorders visits the clinic.

**Study period and design**: A cross-sectional observational study was conducted between November 2019 and April 2020. All 30 patients who were age ≥18 years with confirmed diagnosis of multiple sclerosis as per 2017 Revised McDonald Criteria (1), gives written consent, and had at least one brain and spinal MRI determined were included in the study.

**Data collection tool and procedure**: A structured questionnaire was used in assessing the demography and clinical characteristics of MS patients. All the patients were clinically evaluated and questionnaires were administered to each participant by certified neurologists. Imaging data were interpreted by a certified Neuroradiologist using a standard format, containing detail characterization of MS-related lesions in terms of their neuroanatomical locations, morphology, number, and symmetry. In addition, complications such as corpus callosal and cortical atrophy were documented. All the MRIs were done using MS protocol. Patient's medical recorders were reviewed for additional clinical data. Out of the thirty patients, 26 had an established multiple sclerosis diagnosis while four was CIS cases. Total of 30 MS patients were currently having followed at the clinic and all of them were diagnosed to have MS in the past five years. However, because of COVID 19 pandemic, only 16 of them visited the clinic for their regular follow up during the study period; and we contacted the rest of the patients via telephone to fill the questionnaires. The detailed clinical characteristics, electrophysiological and other laboratory investigations were reported in the previous unpublished author's manuscript.

**Data analysis**: Completed questionnaires were cleaned and entered to Statistical Package for Social Sciences Version 25 for analysis. Descriptive statistics, chi-square, univariate and multivariate logistic regression analysis are used to describe our findings.

**Ethics approval**: The study received ethical approval from City Government of Addis Ababa Health Bureau Ethical Clearance Committee (Protocol number: A/A/HB/437/227). All subjects provided written and verbal consents before the interview.

## Results

**Demographics and clinical characteristics of study participants**: Thirty patients, 26 were having an established MS and 4 had CIS diagnosis, were included in the present study. Overall, the mean (1SD) age of the study participants was 34.7 (8.9) years (range: 19 – 60 years). Female accounted for majority of the study participants (n=26, 86.7%). The average duration of illness was 3.4 (3.1) years (range: 1 – 11 years). We have compared the baseline characteristics based on age groups (age 35 years and below and above age 35 years). There was significant difference between the mean age of the two age groups (p<0.0001). Similarly, no difference in average duration of illness was observed between the two age groups (p=0.42). Relapsing and remitting MS (n=20, 66.7%) was the commonest type of MS, followed by primarily progressive MS (n=4, 13.3%) and clinically isolate syndrome (n=4, 13.3%). No difference was observed regarding types of MS in the two age groups (p=0.42).

Sixteen (n=30, 53.3%) have undergone electrophysiological studies; of which eleven had a normal nerve conduction study (NCS); five (16.6%) had visual evoked examination; all were abnormal (prolonged P100 latency); and one patient had a normal brain stem auditory evoked test. In addition, 16.6% of the study participants had a cerebrospinal fluid (CSF) analysis and none had an oligoclonal band test from their CSF samples. In the present study, seven patients had diabetes mellitus, two had hypertension, and one had HIV infection. Each of the four (13.3%) participants each reported alcohol use and history of head injury. None of the participants reported history of cigarette smoking and one (3.3%) had history of living in the temperate region (Table 1). Steroid is the commonest prescribed medication for the patients. Two patients received Rituximab and one received Azathioprine.

**Table 1:** Baseline characteristics of the study participants, Yehuleshet Specialty Clinic 2019/2020

Characteristics	Age ≤ 35 years n=18 (60%)	Age > 35 years n=12 (40%)	Total n=30 (100%)	p-value
Age in years (mean, 1SD)	29.4 (4.6)	42.7 (7.9)		0.0001
Male	2 (11.1)	2 (16.7)	4 (13.3)	0.53
Female	16 (88.9)	10 (83.3)	26 (86.7)	
Duration of illness (mean, 1SD)	3.1 (2.9)	4 (3.4)		0.42
MS variants				
Clinical isolate syndrome	4(22.2)	0 (0)	4 (13.3)	0.15
Relapsing and remitting MS	12 (66.7)	8 (66.7)	20 (66.7)	
Secondary progressive MS	0 (0)	2 (16.7)	2 (6.7)	
Primarily progressive MS	2 (11.1)	2 (16.7)	4 (13.3)	
Risk factors of MS				
Living in temperate zone	0 (0)	1 (8.3)	1 (3.3)	0.40
Cigarette smoking	0 (0)	0 (0)	0 (0)	**
Alcohol use	2 (11.1)	2 (16.7)	4 (13.3)	0.51
Head injury	1 (5.5)	3 (25)	4 (13.3)	0.16

¶**No comparison group, MS: Multiple sclerosis, 1SD: one standard deviation

**Figure 1:**
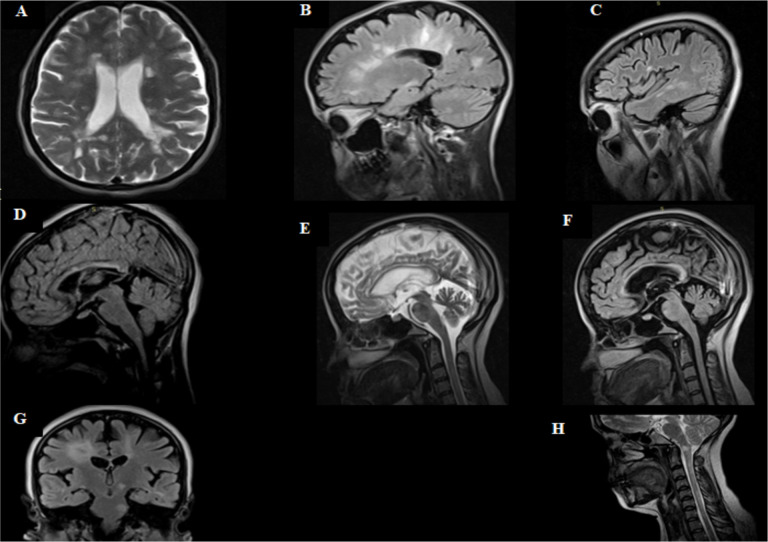
Axial T2 brain MRI showing bilateral periventricular white matter oval/round hyperintense lesions (A). Sagittal FLAIR MRI showing multiple periventricular demyelinating plaques extending radially away from the body of the lateral ventricle, Dawson's fingers (B). Sagittal FLAIR MRI showing multiple juxtacortical lesions abutting the cortex (C). Sagittal FLAIR MRI showing linear hyperintense lesion in the corpus callosum at the callososeptal interface (D). Sagittal T2 weighted image showing corpus callosal atrophy (E). Sagittal FLAIR MRI showing global brain atrophy (F). Coronal FLAIR MRI showing left cerebral peduncle and left pontine nodular hyperintensity (G). Sagittal T2 weighted image showing Rounded hyperintensity at the cervicomedullary junction and long segment T2 hyperintensity in the upper cervical cord.

**Magnetic resonance imaging features of study participants**: We characterize the brain, cervical, and thoracic cord MRI features of 26 patients with established MS and four clinically isolate syndrome. MS-related MRI lesions were characterized based on their intensity in different MR-sequences, neuroanatomical locations, symmetry, morphology, and number. The detailed descriptions are mentioned in table 2. All the patients had multiple, ovoid, and symmetrical lesions which are T_2_ hyperintense and T_1_ hypointense (Figure 1D). Lesions were observed in the periventricular region in all thirty patients, whereas, the subcortical white matter region (n=28, 98.3%) and corpus callosum (n=21, 70%) were involved subsequently respectively. Cervical cord was affected in 40% of the patients (n=12) (Figure 1C & 1F). Out of thirty patients, 16.6% (n=5) had one or more post contrast enhancement. However, none of the lesions in all the patients had perilesional edema. Dawson's fingers projections were observed in 40% of the patients (n=12). No difference was observed regarding Dawson's projections between the two age groups (p=0.41) (Figure 1A, 1B & 1E).

**Table 2:** MRI features of the study participants, Yehuleshet Specialty Clinic, 2019/2020

Neuroimaging findings	Age ≤ 35 years n=18 (60%)	Age > 35 years n=12 (40%)	Total n=30 (100%)	p-value
Periventricular lesions	18 (100%)	12 (100)	30 (100)	**
Juxtacortical lesion	10 (55.5)	4 (33.3)	14 (46.7)	0.21
Subcortical lesions	17 (94.4)	11 (91.7)	28 (93.3)	0.65
Midbrain lesions	5 (27.8)	2 (16.7)	7 (23.3)	0.40
Pontine lesions	8 (44.4)	5 (41.7)	13 (43.3)	0.59
Medulla lesions	0 (0)	1 (8.3)	1 (3.3)	0.40
Cerebellar peduncles lesions	6 (33.3)	1 (8.3)	7 (23.3)	0.13
Cerebellar hemispheres lesions	1 (5.6)	2 (16.7)	3 (10)	0.35
Corpus callosum lesions	16 (99.9)	5 (41.7)	21 (70)	0.009
Pericallosal lesions	14 (77.8)	5 (41.7)	19 (63.3)	0.04
Atrophied corpus callosum	2 (11.1)	0 (0)	2 (6.7)	0.35
Dawson's projections	8 (44.4)	4 (33.3)	12 (40)	0.41
Cervical spinal cord lesions	8 (44.4)	4 (33.3)	12 (40)	0.41
Thoracic spinal cord lesions	1 (5.6)	1 (8.3)	2 (6.7)	0.65
U-fiber lesions	3 (16.7)	4 (33.3)	7 (23.3)	0.27
Global brain atrophy	4 (22.2)	1 (8.3)	5 (16.7)	0.32

** No comparison group, n=frequency

1. *Periventricular lesions:* T2-hyperintense cerebral white matter lesion in direct contact with the lateral ventricles, without intervening white matter

2. *Pericallosal lesions:* T2-hyperintense white matter lesions involving around corpus callosaum

3. *U-fiber lesions:* T2-hyperintense white matter lesion involving U-fiber

4. *Juxtacortical lesions:* T2-hyperintense white matter lesion abutting the cortex without intervening normal white matter

5. *Subcortical lesions:* T2-hyperintense white matter lesion seen in areas other than regions mentioned from 1–4.

*Massimo F. et al, 2019*

**Figure 2:**
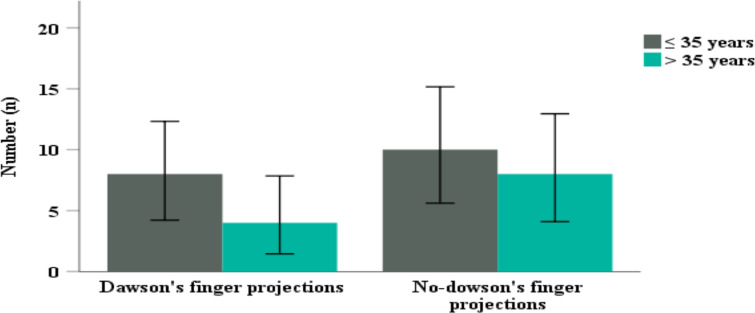
Distribution of Dowson's finger projections in MS patient's by age.

**Figure 3:**
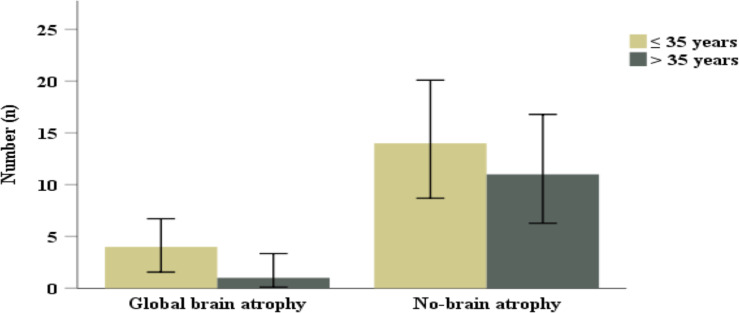
Distribution of global brain volume loss in MS patients by age.

Half of the patients had juxtacortical area lesions (n=14, 46.7%). Among posterior fossa structures pons was the most affected (n=13, 43.3%), followed by cerebellar peduncles (n=7, 23.3%). Lesions in the corpus callosum and pericallosal region showed significant difference among the two age groups (p=0.009, p=0.04, respectively). Corpus colossal atrophy and global brain volume loss was observed in 6.7% (n=2) and 16.7% (n=5) respectively. Lesions affecting a short association fiber such as U-fibers was observed in 23.3% (n=7) of the participants. Dowson's finger projections were observed more in those age 35 years and below compared to those age > 35 years without reaching a statistical significance (p=0.41) (Figure 2). Similarly, global brain atrophy was observed among those age 35 years and below compared to those age > 35 years (p=0.62) (Figure 3).

**Relapsing and remitting MS and covariates—univaariate and multivariate analysis**: Multivariate analysis showed increase in duration of illness was associated with RRMS type when adjusted for age and history of head injury (AOR 1.64, 95% CI1.01–2.67, p=0.04). However, age and history of head injury were not found to be predictors of RRMS type (Table 3). Similarly, age was found to be independent predictor of MS-associated lesions involving corpus callosum. Both univariate and multivariate analysis showed similar results regarding association between age and corpus colossal involvement. For every one-year increment in the age of the patient, the risk of having corpus callosal lesions increase by 13% (AOR 1.13, 95% CI 1.01–1.28, p=0.04). Duration of illness and history of head injury were not found to be predictors of corpus callosum lesions (Table 3).

**Table 3:** Univariate and multivariate analysis, Yehuleshet Specialty Clinic 2019/2020

	Relapsing and Remitting multiple sclerosis					
Variables	COR	95% CI	p-value	AOR	95% CI	p-value
Duration of illness	1.47	0.98–2.22	0.06	1.64	1.01–2.67	0.04
Age	0.97	0.89–1.06	0.5	0.94	0.85–1.05	0.3
Head injury history	0.44	0.05–3.74	0.4	0.21	0.01–3.29	0.3
	Corpus callosum lesions					
Duration of illness	1.11	0.86–1.42	0.42	1.08	0.81–1.43	0.6
Age	1.14	1.01–1.30	0.03	1.13	1.01–1.28	0.04
Head injury	2.71	0.32–23.14	0.36	2.24	0.18–27.25	0.5

¶ COR: Crude odds ratio, AOR: Adjusted odds ratio, CI: Confidence interval

## Discussion

To our knowledge this is the first study to describing imaging characteristics of MS patients in Ethiopia. The overall average age was 34.7 years and females accounted for close to 90% of the participants, which is similar to reports from the western countries where MS is more prevalent (3,17). Detailed descriptions of the MRI findings were extracted in terms of dissemination in space (DIS) and dissemination in time (DIT) to fulfill the 2017 McDonald Criteria (1). In addition, MS-specific neuroimaging practical guideline (11) was used as a reference to differentiate MS lesions neuroimaging MS mimickers such as HIV infection, small vessel diseases (SVD) and nonspecific white matter changes associated with hypertension and diabetes mellitus. Alcohol use and head injury were the commonest risk factors identified among our patients. However, only one patient reported history of living in temperate region. These findings support the need to investigate the potential genetic and environmental etiologies predisposing Ethiopian patients to MS (18). Diagnosis of MS is predominantly clinical; using a 2017 McDonald Criteria. Thus, the combination of the typical clinical features, imaging evidences of dissemination in space and/or time, and/or the presence of supportive evidences such as abnormal visual, auditory, and the positive oligoclonal band in CSF (1).

Relapsing and remitting type of MS was the commonest variant in our study participants. This is in line with previously reported studies (4,19,20). Periventricular and subcortical areas were predominantly involved with no difference between the two age groups. Age was found to be independent predictor of lesions involving corpus callosum. Multiple sclerosis symptoms onset is rare before puberty or after the age of 60 years (19). In our cohort the age range from 19 to 60 years, these indicates similarity in demography of MS patients in low and high prevalence region (3).A multiple sclerosis lesion is defined as an area of focal hyperintensity on a T_2_-weighted (T_2_, T_2_-FLAIR or similar) MRI of the CNS. Typical MS lesions are round to ovoid in shape and disseminated in time and place (11). Similarly, all our patient's MRI lesions were T_2_ and FLAIR hyperintense and T_1_ hypointense lesions, with/without contrast enhancement with no perilesional edema. The lesions are multiple, rounded/oval in shape and symmetrically distributed in both hemispheres. Dawson's finger projection, juxtacortical lesions, and short segment spinal cord lesions are said to be hallmark MS lesions (8,11). In the present study half of the patients had juxtacortical lesions. Meanwhile, nearly half of our patients had concomitant cervical and thoracic cord involvement. These findings suggests neuroradiological similarities between MS patients living in the tropics and elsewhere (8–10,11). Infratentorial structures are also prone to MS lesions and in the current study infratentorial structures are affected in up to quarter of the patients. Pontine region was the commonest to be affected in the present study. These findings were consistent with recent review indicating pontine region close to medial longitudinal fascicles are prone to MS lesion because of high concentration of myelinated fibers (11).

The corpus callosum consists of >200 million axons in the adult brain, a majority of which are myelinated. Thus, corpus callosum is significantly affected by MS lesions (11,22). In the present study, nearly two-third had MS lesions in corpus callosum and Pericallosal region, which is in line with previous reports (11,22). Furthermore, among the thirty patients, less than 10% had atrophy of corpus callosum. Since there are no local data for comparison, we have compared this finding with study done in France, which reported severe to moderate corpus callosum atrophy in 60% of their patients. This major discrepancy could be explained by the relatively older patients were enrolled in the French study compared to ours (23).Furthermore, they used 0.5 T MRI, while our MRI machine resolution was only 0.35 T. In the present study, global brain volume loss was observed in less than a quarter of the patients. In addition, global brain atrophy was observed among those patients age 35 years and below compared to those older than age 35. This is likely due to progressive demyelination, wallerian degeneration, and gliosis associated with MS pathogenesis (11,18,24).This finding will support the need for future cognition assessment of our MS patients (21,25,26).

In our study, age was linearly associated with lesions of corpus callosum. This could be explained by the relative susceptibility of corpus callosum to age related changes and MS-related demyelinating lesions. This finding was supported by previous studies, which showed increasing duration of illness was associated with RRMS sub type (11,22,23). Dawson's fingers projection were considered as a hallmark of MS lesion, because of their perpendicular projections towards lateral ventricles (6,11,27), 40% of the patients had this typical MS lesion. In addition, Dowson's projections were observed more in the younger age group compared to the older age group. This finding was lower than recent report from China where they reported Dowson's projection in 73.9% of the study participants (27). This could be likely attributable to utilization of more advanced MRI machines and large number of patients. Limitations of this study include absence of controlled group for comparison and relatively small sample size due to low prevalence of the disease.

In conclusion, typical neuroimaging features of MS were prevalent among Ethiopian MS patients. Age was an independent predictor of lesions involving corpus callosum. Brain atrophy was common among MS patients in Ethiopia.
